# Temporal changes in plasma choline and choline metabolite concentrations in response to an esophageal bolus of rumen-protected fish oil in early lactation cows fed rumen-protected choline

**DOI:** 10.3168/jdsc.2025-0880

**Published:** 2026-01-16

**Authors:** Víctor Sáinz de la Maza-Escolà, Maria Federica Marchesi, Michele Dei Cas, Sara Casati, Fiorenzo Piccioli-Cappelli, Erminio Trevisi, Andrea Piva, Ester Grilli, Joseph W. McFadden

**Affiliations:** 1 Department of Veterinary Medical Sciences, University of Bologna, Bologna, 40064, Italy; 2 Department of Animal Science, Cornell University, Ithaca, NY 14853; 3 Department of Health Sciences, University of Milan, Milan, 20142, Italy; 4 Department of Biomedical, Surgical and Dental Sciences, University of Milan, Milan, 20122, Italy; 5 Fondazione IRCCS Ca' Granda Ospedale Maggiore Policlinico, Milan, 20122, Italy; 6 Department of Animal Sciences, Food and Nutrition, Faculty of Agriculture, Food and Environmental Science, Università Cattolica del Sacro Cuore, Piacenza, 29122, Italy; 7 Vetagro S.p.A., Reggio Emilia 42124, Italy; 8 Vetagro Inc., Miami, FL 33130

## Abstract

Fish oil (FO) is a rich source of n-3 fatty acids (FA), which may be preferable FA for hepatic phosphatidylcholine (PC) synthesis via the actions of phosphatidylethanolamine *N*-methyltransferase. Our objective was to determine whether feeding rumen-protected choline (RPC) increased concentrations of circulating PC enriched with n-3 FA in cows fed a bolus of rumen-protected FO. Eighteen Holstein cows (3.6 ± 2 [mean ± SD] lactations) were randomly assigned to 1 of 2 groups (n = 9/group) at −21 ± 3 d prepartum: unsupplemented (control [CON]) or supplemented with RPC (RPC; 0 or 60 g/d, respectively; 25% choline chloride; Ruprocol, Vetagro S.p.A., Reggio Emilia, Italy) until d 35 postpartum. At d 27 ± 4 postpartum, all cows were provided with a gelatin capsule containing 100 g of lipid-microencapsulated FO as an esophageal bolus (36% FO; 10.2 g of n-3 FA; Prototype 6, Vetagro S.p.A.). Cows were fed a TMR and milked twice daily. Blood was sampled at 0, 10, and 24 h relative to bolus delivery. Plasma was analyzed for choline and choline metabolites using liquid chromatography tandem MS. Data were analyzed under a mixed model with the random effect of cow and the fixed effects of treatment, hour, and their interaction. At h 0, plasma betaine concentrations were greater in RPC relative to CON; however, plasma choline concentrations were not modified by treatment. Following FO bolus administration, circulating trimethylamine *N*-oxide and dimethylglycine (DMG) were increased by h 10. Cows supplemented with RPC tended to have greater plasma DMG and methionine relative to CON. Although no changes were detected for plasma lysophophatidylcholine (LPC)-22:5, LPC-22:6, total LPC, PC-16:0/22:5 or PC-18:0/22:5, the RPC group had increased plasma LPC-20:5, PC-16:0/22:6, and PC-18:0/22:6 compared with CON. Plasma PC-18:0/20:5 and total PC concentrations were greater in RPC cows relative to CON by h 24. We conclude that RPC feeding augmented the ability of dietary FO to increase plasma PC concentrations in early-lactation cows.

## Graphical Abstract

**Figure:**
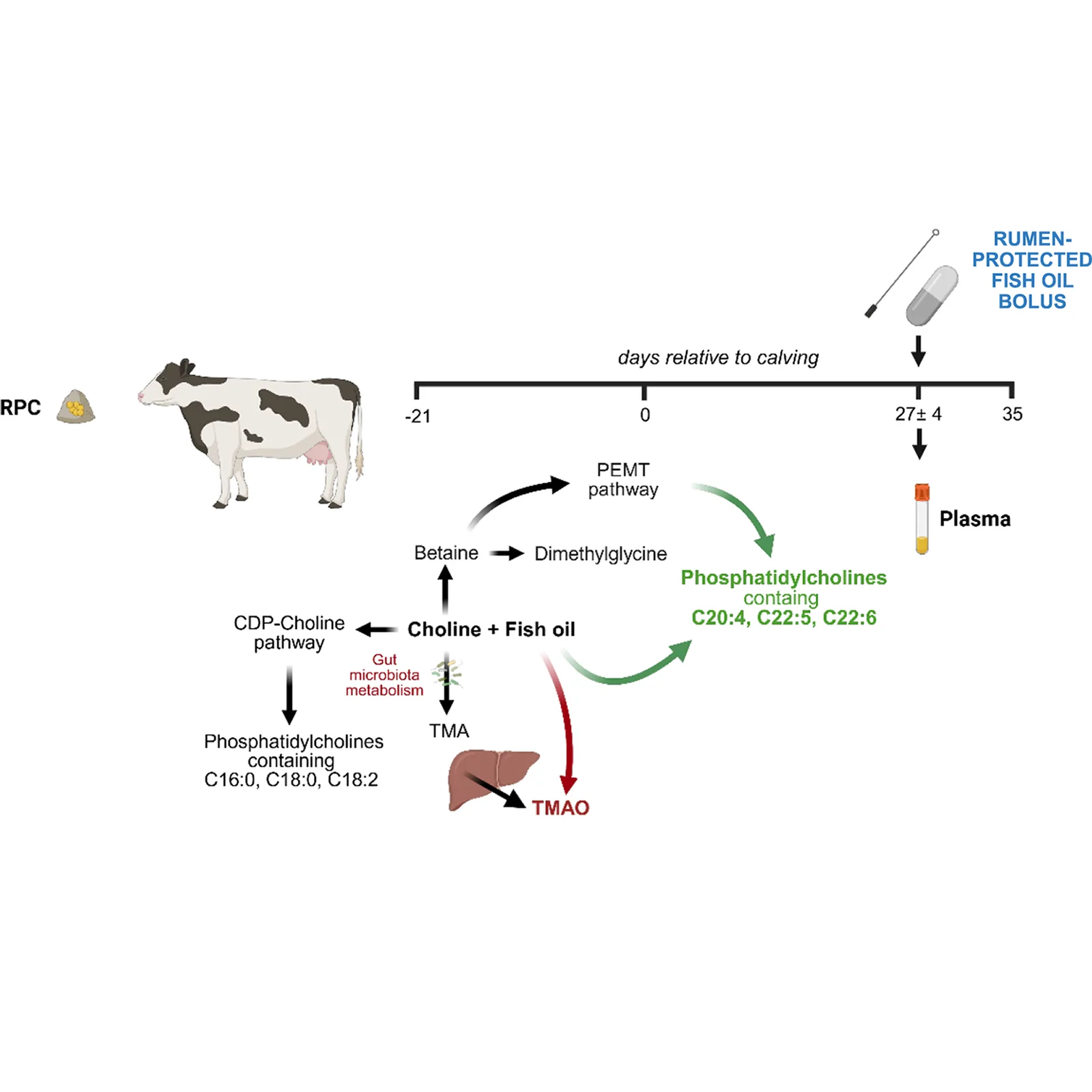

Postpartum hepatic steatosis in dairy cattle remains a nutrition and management challenge, affecting up to 30% to 50% of multiparous cows and being associated with decreased health, fertility, and milk performance (Bobe et al., 2004). Accumulation of hepatic triacylglycerol (**TAG**) has been linked with reduced gluconeogenesis and heightened inflammatory responses (Sordillo et al., 2009), whereas prolonged inflammation during the transition period exacerbates hepatic lipid deposition (Bertoni et al., 2008). Very low-density lipoproteins (**VLDL**) transport TAG from the liver to peripheral tissues; however, compared with nonruminants, dairy cows have limited ability to export TAG as VLDL (Pullen et al., 1990). Consistently, plasma TAG and TAG-rich lipoproteins decrease markedly from gestation to lactation (Davis et al., 2018; Saed Samii et al., 2018).

Phosphatidylcholine (**PC**), the main glycerophospholipid of VLDL, is synthesized through the cytidine diphosphate (**CDP**)-choline pathway and the phosphatidylethanolamine *N*-methyltransferase (**PEMT**) pathway (McFadden et al., 2020). Feeding rumen-protected choline (**RPC**) is a common dietary strategy in peripartum dairy cows to support PC synthesis (Myers et al., 2019; Arshad et al., 2020). The CDP-choline pathway uses choline as a precursor, whereas the PEMT pathway relies on S-adenosylmethionine (**SAM**) as the methyl donor to methylate phosphatidylethanolamine (**PE**). The PEMT enzyme is expressed primarily in hepatic tissue (Vance and Ridgway, 1988). Choline also contributes to methyl group supply via its oxidation to betaine (Zeisel, 1990), which donates a methyl group to homocysteine through betaine-homocysteine S-methyltransferase (**BHMT**), regenerating methionine and dimethylglycine (**DMG**). Methionine is then converted to SAM, which serves as the methyl donor for methylation reactions, including PEMT-mediated PC synthesis (DeLong et al., 1999). In bovine hepatocytes, choline supplementation upregulated genes involved in transmethylation (BHMT, methionine synthase, and methylenetetrahydrofolate reductase), suggesting its contribution to methionine remethylation (Chandler and White, 2017). Methionine, betaine, and folic acid are other methyl donors involved in one-carbon metabolism, with methionine being a preferred substrate for the PEMT pathway (Zhou et al., 2018)

Beyond the role of choline in PC synthesis, the fatty acyl composition of PC influences hepatic lipid metabolism. Saed Samii et al. (2017, 2018) showed that transition cows with high hepatic lipid content (i.e., >10% TAG; Bobe et al., 2004) have reduced concentrations of highly unsaturated PC species. In nonruminants, PEMT preferentially methylates PE enriched in very-long-chain (**VLC**) n-3 FA, such as eicosapentaenoic acid (C20:5n-3; **EPA**) and docosahexaenoic acid (C22:6n-3; **DHA**), whereas the CDP-choline pathway prefers diacylglycerol-containing saturated and monounsaturated FA such as palmitic (C16:0) and oleic acids (*cis*-9 C18:1; DeLong et al., 1999). These FA, largely mobilized from adipose tissue during negative energy balance, are associated with hepatic TAG accumulation (Contreras et al., 2010). Abomasal infusion of algae oil rich in DHA enhanced the incorporation of DHA and other PUFA into PC in lactating cows relative to cows infused with PA (Myers et al., 2019). Circulating lysophophatidylcholines (**LPC**; a form derived from PC turnover) and PC containing PUFA, including DHA and EPA, are typically low in transition cows and indicative of lipid accumulation (Rico et al., 2021). In our previous work (Rico et al., 2025), an esophageal bolus of rumen-protected fish oil (**FO**) rich in EPA and DHA increased plasma VLC n-3 FA and LPC and PC containing PUFA, confirming preferential PUFA incorporation within phospholipids (Stamey et al., 2012). In addition, both EPA and DHA also exert anti-inflammatory effects through direct and indirect inhibitory mechanisms (Lee et al., 2010), adding further physiological relevance to their supply during early lactation.

Microbial degradation of choline to trimethylamine (**TMA**) can occur in the rumen or intestines, and the absorbed TMA is then rapidly oxidized in the liver to trimethylamine N-oxide (**TMAO**; Zeisel et al., 1983), thereby potentially limiting the bioavailability of dietary choline. Fish oil naturally contains TMAO (Lombardo et al., 2021), and increases in circulating TMAO have also been observed following abomasal choline infusion, dietary lecithin (a source of PC), or supplementation with RPC in dairy cows (Myers et al., 2019; Wang et al., 2021; France et al., 2022). In humans, higher TMAO concentrations are associatively linked with metabolic disease (León-Mimila et al., 2021), although causality remains unclear. Importantly, intravenous TMAO infusion in early-lactation dairy cows did not impair metabolic health (Myers et al., 2021), indicating species-specific differences in the physiological relevance of circulating TMAO.

The supply of PUFA in dairy cows is limited because of rumen biohydrogenation, feeding FO in a rumen-protected form is a means to provide EPA and DHA postruminally. Therefore, co-supplementation of RPC and rumen-protected FO in transition cows could enhance PEMT activity and PC synthesis and thus support the secretion of TAG within VLDL. Our objective was to quantify changes in circulating polyunsaturated LPC and PC, as well as choline metabolites, in response to an esophageal bolus of rumen-protected FO in lactating dairy cows supplemented with RPC.

A total of 18 early-lactation multiparous Holstein dairy cows (3.6 ± 2 lactations; 2.7 ± 0.3 BCS) at the Università Cattolica del Sacro Cuore Research Dairy Farm (Piacenza, Italy) were used in this study. The cows were part of a larger study that evaluated the effects of feeding RPC from −21 d prepartum to 35 d postpartum (Sáinz de la Maza-Escolà et al., 2025). In a complete randomized design, cows were assigned to 1 of 2 groups (n = 9/group); control (**CON**), or TMR supplemented with 60 g/d of RPC (lipid-microencapsulated choline chloride 25%; Ruprocol, Vetagro S.p.A., Reggio Emilia, Italy; RPC). The RPC supplement was mixed daily into the TMR, and cows were kept in a common pen, had free access to water, and were individually fed ad libitum using the Roughage Intake Control system (RIC, Hokofarm Group, AXE Emmeloord, the Netherlands; 3 feeders/treatment in pre- and postpartum pens). The diet met NASEM (2021) requirements and contained 32.5% amylase-treated NDF expressed on an OM basis, 16.5% CP, 3.85% ether extract, and 7.54% ash. Cows were milked at 0400 and 1600 h, and milk yield and components were recorded daily. Production and blood results from this study are reported in Sáinz de la Maza-Escolà et al. (2025).

At d 27 ± 4 (mean ± SD), postpartum cows received a 100-g esophageal bolus (in a gelatin capsule) of lipid-microencapsulated FO (36% FO; Prototype 6, Vetagro S.p.A., Reggio Emilia, Italy). The bolus was provided at 0900 immediately after TMR offering. The FO prototype was manufactured using a patented microencapsulation technique that protects nutrients and other active compounds from ruminal degradation, yet releases them for absorption in the small intestine. This prototype was previously tested by Rico et al. (2025). The hydrogenated palm oil matrix used in this prototype is similar to the matrix used in other Vetagro products. The FO bolus delivered 10.2 g, equivalent to the sum of EPA (6.8%) and DHA (4.4%), and contained less than 1% of docosapentaenoic acid (22:5 n-3). Blood samples were collected in EDTA-containing tubes (Covidien, Mansfield, MA) at 0, 10, and 24 h relative to bolus administration via jugular venipuncture. These time points were selected based on Rico et al. (2025), who showed that circulating EPA- and DHA-derived metabolites from rumen-protected lipid boluses peak between 8 and 12 h. For separation of EDTA-containing plasma, blood samples were placed on ice for 30 min followed by centrifugation at 3,400 × *g* for 15 min at 4°C. Plasma was stored at −20°C until analysis. Total mixed ration samples were analyzed for nutrient composition using standard procedures (AOAC International, 2012). Milk fat, protein, and lactose of each cow was recorded at each milking by an inline analyzer (Afimilk Ltd., Kibbutz Afikim, Israel).

All plasma metabolites were measured using liquid chromatography tandem MS. Analyses were performed on a 1290 Infinity ultra-high-performance liquid chromatography system (Agilent Technologies, Palo Alto, CA) coupled to a Q Trap 5500 linear ion trap triple quadrupole mass spectrometer (Sciex, Darmstadt, Germany) and equipped with an electrospray ionization source in a positive mode. Sample preparation for LPC and PC was conducted according to the method developed by Dei Cas et al. (2020). Plasma choline, methionine, betaine, DMG and TMAO were extracted according to the method developed by Steuer et al. (2016). All compounds were monitored in multiple reaction monitoring mode. For quantitative measurements, the area of the signals of each compound was normalized to that corresponding deuterium isotopes used as internal standards in each analytical run.

Baseline plasma data were analyzed using a general mixed model including random effect of cow, and fixed effects of DIM, parity, and treatment. All plasma time points data were analyzed as repeated measures over time relative to FO bolus administration under a mixed model including random effect of cow and fixed effects of DIM, parity, baseline measurement, and treatment, time and their interaction. The fixed effect of DIM and parity were tested but removed from the model because *P* > 0.30. We removed 5 individual observations across all metabolites (<2% of total observations), all based on prespecified Studentized residual criteria (>|3|). Tukey's post hoc test was used for multiple comparison correction of *P*-values for all pairwise comparisons of LSM. Significance was declared at *P* ≤ 0.05 and tendencies were declared at 0.05 < *P* ≤ 0.15. All statistics were performed using the statistical software JMP Pro v. 16.2 (SAS Institute Inc., Cary, NC).

No differences (*P* > 0.15) were detected among treatments for DMI (24.4 ± 2.1 kg/d; mean ± SD), milk yield (46.5 ± 5.8 kg/d), content and yields of milk fat (4.34% ± 0.3% and 1.91 ± 0.26 kg/d), protein (3.25% ± 0.34% and 1.44 ± 0.28 kg/d), and lactose (4.72% ± 0.29% and 2.08 ± 0.35 kg/d) on the day following the bolus administration. At h 0 (baseline; Table 1), plasma betaine concentrations were greater in RPC than CON (*P* = 0.05). Plasma DMG (*P* = 0.13) and PC-18:0/22:5 (*P* = 0.10) tended to be higher in RPC than CON. However, plasma choline, methionine, TMAO, and the remaining LPC (LPC-20:5, LPC-22:5, LPC-22:6, and total LPC) and PC (PC-16:0/20:5, PC-18:0/20:5, PC-16:0/22:5, PC-16:0/22:6, PC-18:0/22:6, and total PC) concentrations were not different before the administration of the FO bolus.

**Table 1:** Baseline plasma choline metabolites and polyunsaturated LPC and PC concentrations in early-lactation Holstein cows supplemented with rumen-protected choline (RPC) or not (CON) before the provision of a single esophageal bolus of rumen-protected fish oil

Metabolite,^2^ μg/mL	Treatment^1^		SEM	Treatment *P-*value^3^
	CON	RPC		
Choline metabolites				
Choline	2.81	2.98	0.16	0.41
Methionine	6.11	6.85	0.35	0.16
Betaine	5.09	6.14	0.36	0.05
DMG	3.12	3.80	0.32	0.13
TMAO	7.57	8.99	1.81	0.57
Polyunsaturated LPC				
LPC-20:5	0.24	0.26	0.02	0.49
LPC-22:5	0.68	0.55	0.10	0.31
LPC-22:6	0.07	0.09	0.01	0.41
Total LPC^4^	1.00	0.96	0.07	0.69
Polyunsaturated PC				
PC-16:0/20:5	5.61	6.16	0.81	0.62
PC-18:0/20:5	8.19	6.13	1.27	0.24
PC-16:0/22:5	0.74	0.69	0.14	0.80
PC-18:0/22:5	3.22	3.85	0.25	0.10
PC-16:0/22:6	0.02	0.02	<0.01	0.39
PC-18:0/22:6	1.97	1.95	0.27	0.96
Total PC^5^	19.4	18.6	2.7	0.85

1 Eighteen multiparous early-lactation cows were randomly assigned to 1 of 2 treatments at −21 d prepartum: unsupplemented (CON; n = 9) or supplemented with 60 g/d of RPC (lipid-microencapsulated choline chloride 25%; Ruprocol, Vetagro S.p.A., Reggio Emilia, Italy; RPC; n = 9) and provided with a single esophageal bolus of encapsulated fish oil (36%; Prototype 6, Vetagro S.p.A., Reggio Emilia, Italy) at d 27 ± 4 (mean ± SD) postpartum.

2 DMG = dimethylglycine; TMAO = trimethylamine *N*-oxide; LPC = lysophosphatidylcholine; PC = phosphatidylcholine.

3 Significance was considered when *P* ≤ 0.05. Trend toward significance was considered when 0.05 < *P* ≤ 0.15.

4 Total LPC is the sum of LPC-20:5, LPC-22:5, and LPC-22:6.

5 Total PC is the sum of PC-16:0/20:5, PC-18:0/20:5, PC-16:0/22:5, PC-18:0/22:5, PC-16:0/22:6, and PC-18:0/22:6.

Plasma changes in choline metabolites, LPC, and PC after the single rumen-protected FO bolus administration are depicted in Figure 1, Figure 2. No differences were detected in plasma choline and betaine concentrations. Methionine concentrations tended be lower in RPC cows compared with CON (treatment, *P* = 0.07; Figure 1). Cows fed RPC had greater plasma DMG concentrations compared with CON (treatment, *P* = 0.03), with a time effect (*P* = 0.03) mostly observed at 10 h relative to bolus administration (Figure 1). Plasma TMAO concentrations peaked at h 10 for both CON and RPC cows (time, *P* < 0.01; Figure 1). We did not detect any significant effects for plasma LPC-22:5, LPC-22:6, or total LPC concentrations (Figure 2). However, RPC increased LPC-20:5 concentrations compared with CON (treatment, *P* = 0.03; Figure 2). We observed increases in plasma PC-16:0/20:5, PC-18:0/20:5, and total PC concentrations in RPC cows compared CON (*P* < 0.05; Figure 2), with a treatment × time interaction (*P* = 0.06, *P* = 0.01, and *P* = 0.04, respectively; Figure 2) observed at h 24 relative to bolus administration. Cows supplemented with RPC had greater concentrations of plasma PC-16:0/22:6 compared with CON (treatment, *P* = 0.01), with a time effect (*P* = 0.01) observed at h 24 relative to bolus administration (Figure 2). Plasma PC-18:0/22:6 concentrations tended to be greater for RPC compared with CON (*P* = 0.07; Figure 2). No changes were detected in plasma PC-16:0/22:5 and PC-18:0/22:5 concentrations with treatment (Figure 2).

**Figure 1:**
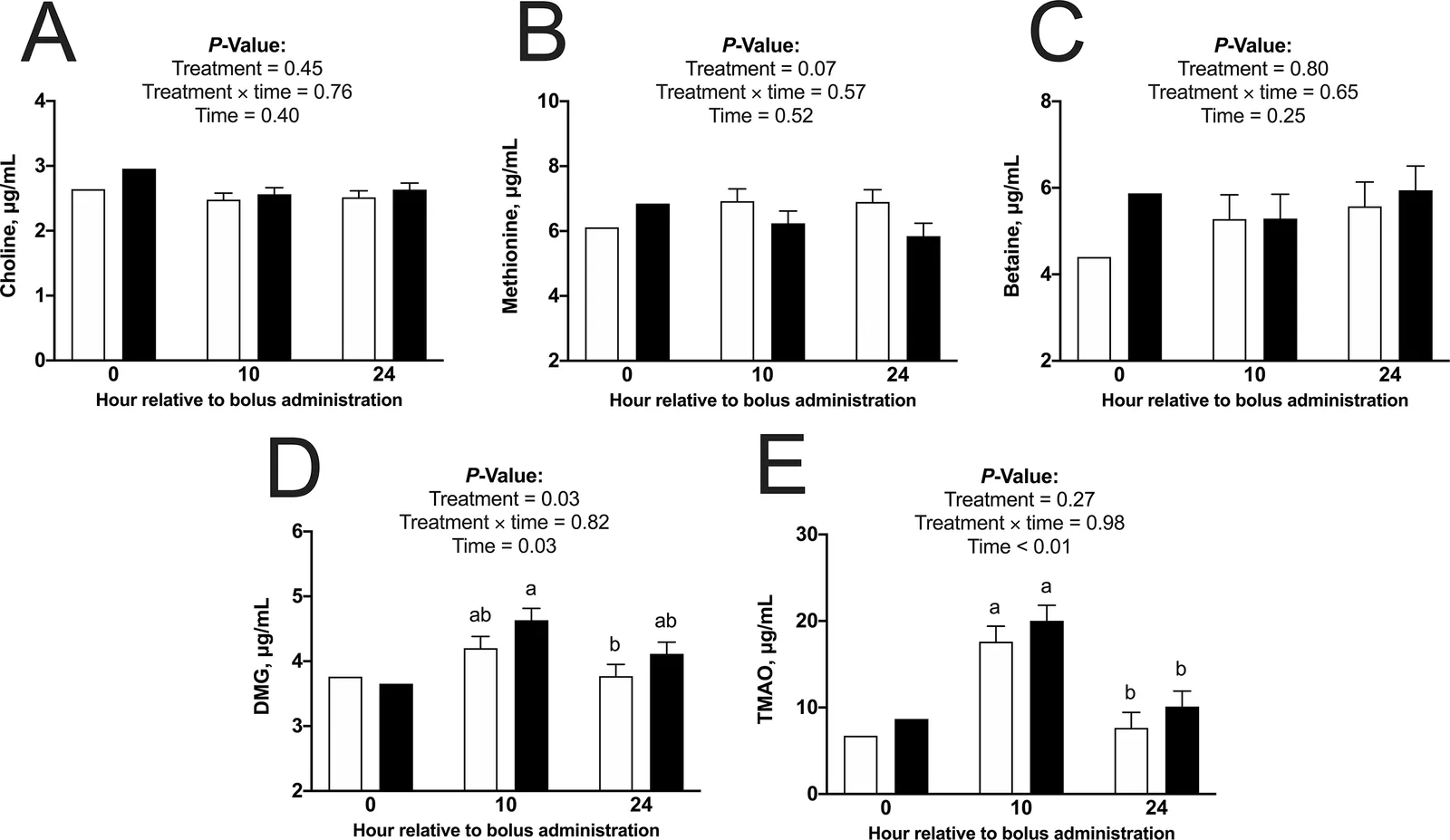
Temporal changes in plasma (A) choline, (B) methionine, (C) betaine, (D) dimethylglycine (DMG), and (E) trimethylamine *N*-oxide (TMAO) concentrations in 18 early-lactation Holstein dairy cows supplemented with rumen-protected choline (RPC; black bar) or unsupplemented (CON; white bar) and provided with a single esophageal bolus of rumen-protected fish oil (36%; Prototype 6, Vetagro S.p.A., Reggio Emilia, Italy). Data are presented as LSM ± SEM. Different lowercase letters (a,b) denote significance (*P* < 0.05).

**Figure 2:**
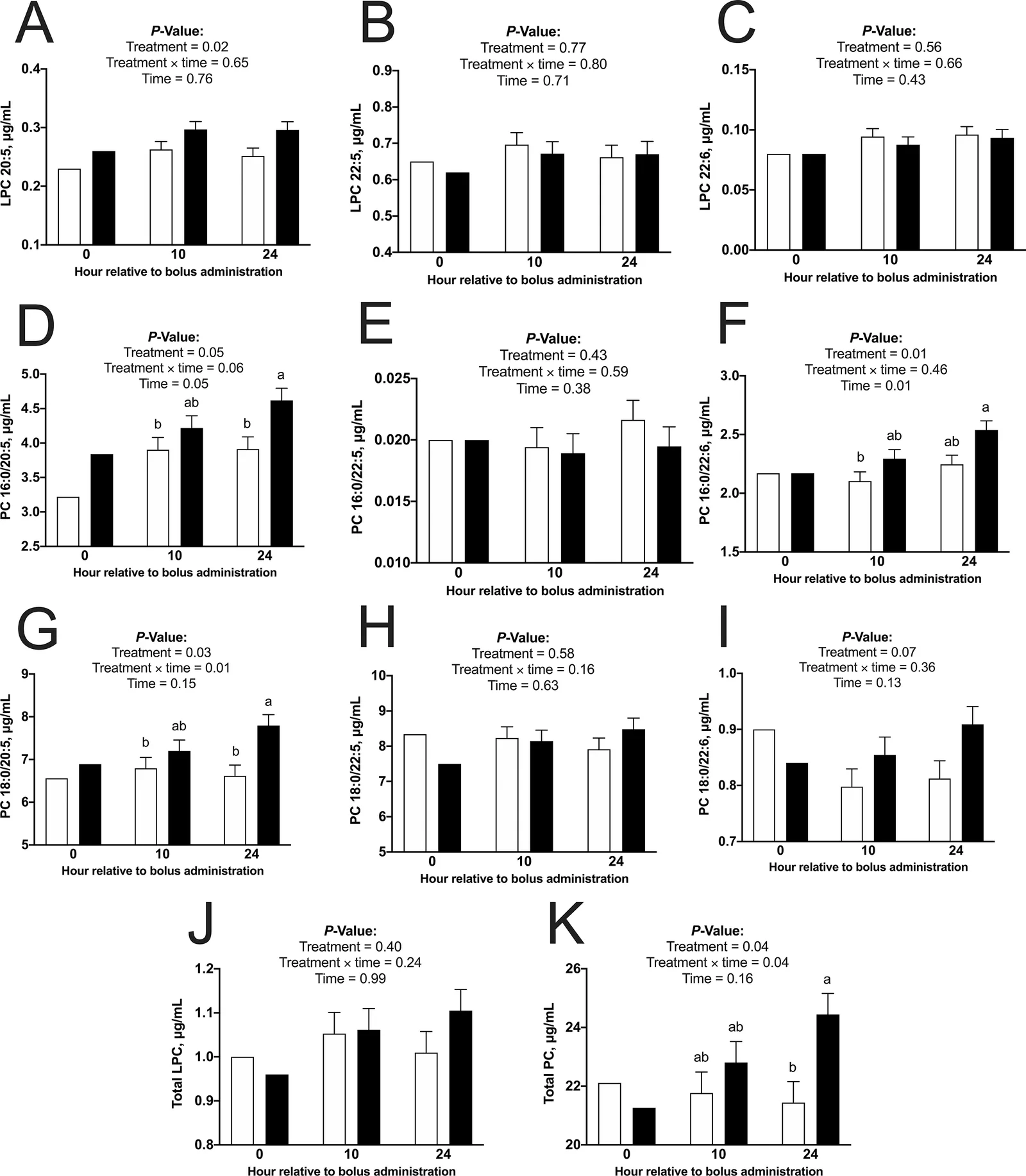
Temporal changes in plasma (A) lysophosphatidylcholine (LPC) LPC-20:5, (B) LPC-22:5, (C) LPC-22:6, (D) phosphatidylcholine (PC) PC-16:0/20:5, (E) PC-16:0/22:5, (F) PC-16:0/22:6, (G) PC-18:0/20:5, (H) PC-18:0/22:5, (I) PC-18:0/22:6, (J) total LPC, and (K) total PC concentrations in 18 early-lactation Holstein dairy cows supplemented with rumen-protected choline (RPC; black bar) or unsupplemented (CON; white bar) and provided with a single esophageal bolus of rumen-protected fish oil (36%; Prototype 6, Vetagro S.p.A., Reggio Emilia, Italy). Data are presented as LSM ± SEM. Different lowercase letters (a,b) denote significance (*P* < 0.05). Total LPC = LPC-20:5 + LPC-22:5 + LPC-22:6. Total PC = PC-16:0/20:5 + PC-18:0/20:5 + PC-16:0/22:5 + PC-18:0/22:5 + PC-16:0/22:6 + PC-18:0/22:6.

The co-supplementation of dietary methyl donors and n-3 FA to promote hepatic PC synthesis is a great opportunity to enhance TAG export in postpartum dairy cows at risk for metabolic disease. Actual nutritional strategies do not consider FA nutrition for these purposes. Our data show that the supplementation of RPC plus the provision of a rumen-protected FO bolus improves the synthesis of polyunsaturated PC in early-lactation dairy cows. This combination also increased the plasma concentration of DMG and tended to lower plasma methionine, overall suggesting greater flux through the transmethylation cycle to supply methyl groups to the PEMT pathway. These results provide evidence to consider FA nutrition to optimize PC synthesis in transition dairy cows.

Before FO provision, RPC-supplemented cows had greater plasma betaine concentrations, a common response when feeding rumen-protected choline (de Veth et al., 2016). In contrast, baseline plasma TMAO levels did not differ between control and RPC cows, indicating negligible microbial degradation of the supplemented choline and supporting its effective bioavailability (France et al., 2022). Although these responses are consistent with partial escape of choline from ruminal degradation, our study did not include urine or fecal measurements; therefore, whole-body choline partitioning cannot be inferred and should be addressed in future work. Regardless of treatment, provision of FO increased circulating TMAO—an analyte inherently present in FO (Lombardo et al., 2021)—and such increases have not been associated with detrimental effects in early-lactation cows (Myers et al., 2021). Future studies should determine the ability of this feeding strategy to enhance hepatic TAG secretion within VLDL and to reduce fatty liver prevalence in dairy cattle.
